# Community-level interventions for pre-eclampsia (CLIP) in Pakistan: A cluster randomised controlled trial

**DOI:** 10.1016/j.preghy.2020.07.011

**Published:** 2020-10

**Authors:** Rahat N. Qureshi, Sana Sheikh, Zahra Hoodbhoy, Sumedha Sharma, Marianne Vidler, Beth A. Payne, Imran Ahmed, J. Mark Ansermino, Jeffrey Bone, Dustin T. Dunsmuir, Tang Lee, Jing Li, Hannah L. Nathan, Andrew H. Shennan, Joel Singer, Domena K. Tu, Hubert Wong, Laura A. Magee, Peter von Dadelszen, Zulfiqar A. Bhutta, the CLIP Pakistan Working Group

**Affiliations:** a Centre of Excellence, Division of Woman and Child Health, Aga Khan University, Stadium Road, P. O. Box 3500, Karachi 74800, Pakistan; b Department of Obstetrics and Gynaecology, Faculty of Medicine, University of British Columbia, Suite 930, 1125 Howe Street, Vancouver, BC V6Z 2K8, Canada; c Centre for International Child Health, University of British Columbia, 305-4088 Cambie Street, Vancouver, BC V5Z 2X8, Canada; d Department of Women and Children’s Health, School of Life Course Sciences, Faculty of Medicine and Life Sciences, King’s College London, 1 Lambeth Palace Road, London SE1 7EH, UK; e Centre for Health Evaluation and Outcome Sciences, Providence Health Care Research Institute, University of British Columbia, 588 – 1081 Burrard Street, St. Paul’s Hospital, Vancouver, BC V6Z 1Y6, Canada; f Centre for Global Child Health, Hospital for Sick Children, 525 University Avenue, Suite 702, Toronto, ON M5G 2L3, Canada; g the CLIP Pakistan Trial Working Group (Table S1)

**Keywords:** Cluster randomized controlled trial, Pregnancy hypertension, Pakistan, Community engagement, Mobile health, Community health worker

## Abstract

**Objectives:**

To reduce all-cause maternal and perinatal mortality and major morbidity through Lady Health Worker (LHW)-facilitated community engagement and early diagnosis, stabilization and referral of women with preeclampsia, an important contributor to adverse maternal and perinatal outcomes given delays in early detection and initial management.

**Study design:**

In the Pakistan Community-Level Interventions for Pre-eclampsia (CLIP) cluster randomized controlled trial (NCT01911494), LHWs engaged the community, recruited pregnant women from 20 union councils (clusters), undertook mobile health-guided clinical assessment for preeclampsia, and referral to facilities after stabilization.

**Main outcome measures:**

The primary outcome was a composite of maternal, fetal and newborn mortality and major morbidity.

**Findings:**

We recruited 39,446 women in intervention (N = 20,264) and control clusters (N = 19,182) with minimal loss to follow-up (3∙7% vs. 4∙5%, respectively). The primary outcome did not differ between intervention (26·6%) and control (21·9%) clusters (adjusted odds ratio, aOR, 1∙20 [95% confidence interval 0∙84-1∙72]; p = 0∙31). There was reduction in stillbirths (0·89 [0·81-0·99]; p = 0·03), but no impact on maternal death (1·08 [0·69, 1·71]; p = 0·74) or morbidity (1·12 [0·57, 2·16]; p = 0·77); early (0·95 [0·82-1·09]; p = 0·46) or late neonatal deaths (1·23 [0·97-1·55]; p = 0·09); or neonatal morbidity (1·22 [0·77, 1·96]; p = 0·40). Improvements in outcome rates were observed with 4–7 (p = 0·015) and ≥8 (p < 0·001) (vs. 0) CLIP contacts.

**Interpretation:**

The CLIP intervention was well accepted by the community and implemented by LHWs. Lack of effects on adverse outcomes could relate to quality care for mothers with pre-eclampsia in health facilities. Future strategies for community outreach must also be accompanied by health facility strengthening.

**Funding:**

The University of British Columbia (PRE-EMPT), a grantee of the Bill & Melinda Gates Foundation (OPP1017337).

## Highlights

• Task-sharing activities to detect and manage pregnancy hypertension can be achieved by CHWs.
• Intervention effects may have been masked by incomplete implementation or weak in-facility care.
• Contact intensity analyses support the WHO eight contact antenatal care model.
• Condition-focused community-based interventions without facility strengthening are inadequate.

## 1 Introduction

The Global Burden of Disease estimates that Pakistan, the world’s sixth most populous country, endures the world’s third highest burden of maternal mortality (348·6/100,000 live births [95% uncertainty intervals (UI) 247·2-447·0]), stillbirth (27·6/1000 births [95% UI 23·1-32·8]), and under-five child mortality (58·0/1000 live births [95% UI 50–69]) [1], [2]. In addition, Pakistan is recognised to have the highest neonatal mortality rate globally [3].

Such adversity is associated with sociocultural factors and health system-related barriers to accessing and receiving the best evidence-based care [4], [5], [6], [7]. Among the barriers, delays in recognition of illness and seeking care contributed to 36% of maternal deaths in 2006 [7]. Formative research in Sindh Province reveals the importance of husbands and mothers-in-law as decision makers in health care utilization [4]. Poor availability of transport, financial constraints, and limited mobility of women are important additional barriers to seeking care.

In response, the government of Pakistan has invested in health strategies and outreach services targeting poor and marginalised populations. The public health sector is comprised of primary-, secondary-, and tertiary-level health facilities, while the private health sector includes informal care providers (i.e., traditional birth attendants and spiritual healers) and formal medical clinics and/or hospitals. The mainstay of rural primary care is a cadre of community health workers (Lady Health Workers [8], LHWs). LHWs (currently almost 100,000) receive formal training for 15 months including didactic course work (3 months) and a 12-month practicum. They provide mainly maternal, newborn and child health (MNCH) preventative services, health promotion and referrals, as needed, to 100–150 households, covering 70% of the rural populations in major provinces. LHWs receive monthly refreshers and replenishment of supplies in the primary care facilities of respective districts. On average, as noted in external evaluation [8], LHWs work on average five hours daily, visit an average 27 households per week and provide advice to an average 22 clients, usually women per week. Their current stipend is PKR 27,000 per month (US$ 173).

Pregnancy hypertension is associated with maternal and perinatal morbidity and mortality risks worldwide, particularly in less-developed countries like Pakistan [9]. There is a general lack of understanding by women of the implications of having, and by health care providers of best evidence-based care of, pregnancy hypertension [4]. The detection and management of pregnancy hypertension is an important component of antenatal care (ANC) attendance [10]; its implementation reflects the quality of care in pregnancy. In resource-constrained environments such as Pakistan, standards against which health care delivery is benchmarked are often basic, such as whether BP or proteinuria were tested at least once in pregnancy, when each of these practices is to be delivered serially over the course of pregnancy [11]. Also, despite national policy, supplies of essential commodities (e.g., magnesium sulphate) are inadequate and few birthing facilities have established protocols for eclampsia management.

A number of innovations have been tested and introduced through the LHW programme, especially targeting perinatal and newborn care and childhood illnesses and immunisations [12], [13], [14], [15]. However, they neither carry blood pressure (BP) measurement devices nor receive training about pregnancy hypertension management.

We hypothesised that training LHWs in community engagement (to raise awareness of, and education about, general pregnancy risks and, specifically, pregnancy hypertension) as well as community-based assessment, triage, and initial treatment of pre-eclampsia would improve maternal and perinatal outcomes. The aim of the Community-Level Interventions for Pre-eclampsia (CLIP) cluster randomised controlled trial (cRCT) in Sindh Province, Pakistan was to reduce maternal and perinatal mortality and major morbidity by 20% or more in intervention (vs. control) clusters, through a community-level intervention to address triage, (initial) treatment, and transport (to facility) of women with pregnancy hypertension.

## 2 Methods

The full protocol has been published (https://clinicaltrials.gov/ct2/show/NCT01911494; Supplementary Appendix 1), and approved by the ethics review committees of the Aga Khan University (AKU, 2590-Obs-ERC-13); University of British Columbia (UBC, H12-03497).

### 2.1 Study setting and trial design

**Fig. 1:**
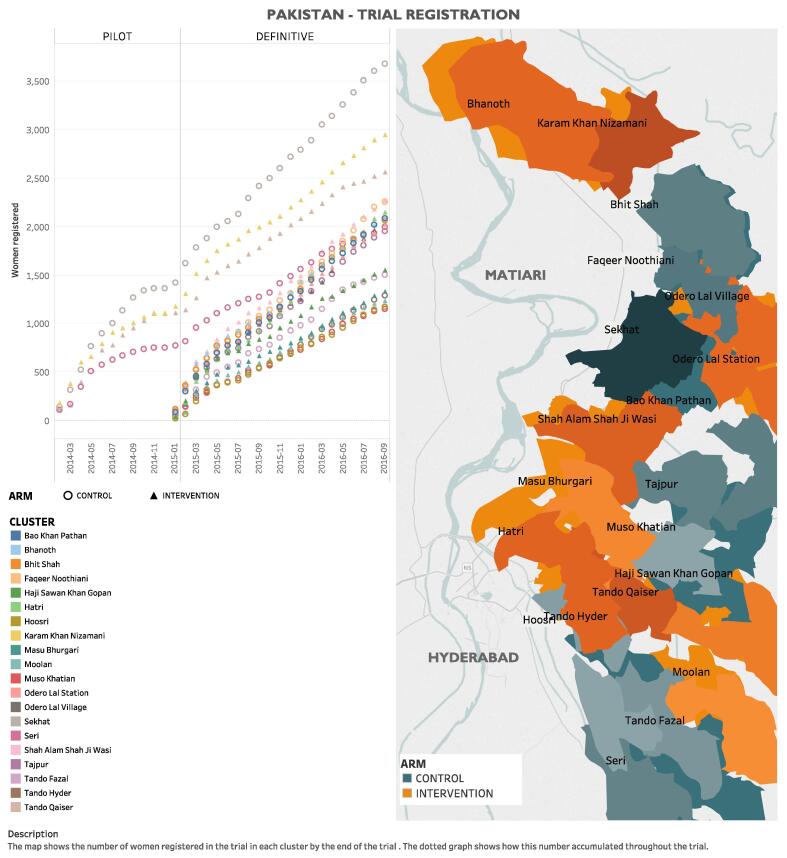
Map of study area and enrollment by cluster.

The CLIP Pakistan trial was one of three independently powered cRCTs (others in India and Mozambique; all NCT01911494), and was conducted in Matiari and Hyderabad districts, Sindh Province, approximately 150 km north of Karachi (Fig. 1). Matiari is rural and located 25 km north of Hyderabad (population: ≈600,000). Hyderabad is semi-urban (population: ≈1,883,000; second most-populous district in Sindh). The consistent population density is 4·7 people/hectare. The vast majority of residents are Muslim, agriculturalists, and speak either Sindhi and/or Urdu. Literacy rates are 40% (Matiari) to 50% (Hyderabad). While women in the study region know about high blood pressure (BP) and seizures in pregnancy, they do not associate the two and they attribute hypertension to stress or weakness [4]. They have no Sindhi term for the most dangerous form of pregnancy hypertension, pre-eclampsia.

The pilot phase (from 7 February 2014 to 18 January 2015), aimed at assessing acceptability of the intervention, and transitioned to the definitive phase (from 19 January 2015 to 31 December 2016) based on ≥50% acceptance by women of health facility referral when indicated by the CLIP intervention (using data to 31 October 2014); no additional outcomes were assessed at the end of the pilot phase. Recruitment of new women ended on 30 September 2016, to allow time for recruited women to deliver by trial end on 31 December 2016.

### 2.2 Participants

Married pregnant women aged between 15 and 49 years were identified by the trial surveillance teams and written informed consent obtained for participation and quarterly data collection in surveillance visits.

### 2.3 Intervention

**Supplementary figure 1:**
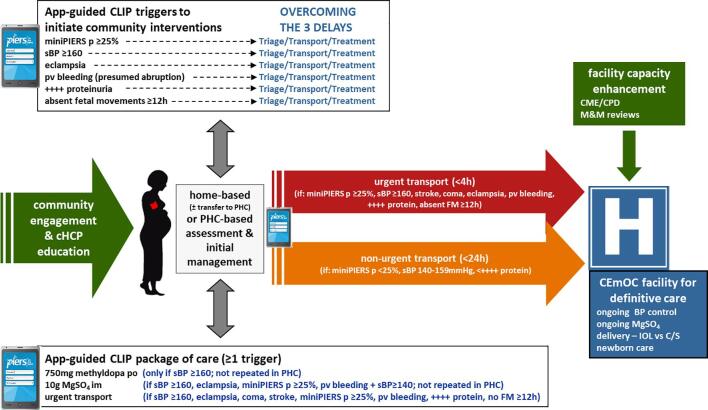
CLIP triggers and treatment.

In the intervention clusters, the CLIP intervention package consisted of an annual group mobilisation meeting with about ten community leaders in each village, and LHW-led visits with eligible women in their homes, with a focus on the detection and management of pregnancy hypertension (Fig. S1). At the first home visit and again at around seven to eight months of pregnancy, LHWs engaged each participant herself, in her home, along with her immediate community (e.g., mother, mother-in-law, and female family or friends as she wished and were available). Activities were supported by culturally-appropriate pictograms developed with the LHW programme and describing maternal and perinatal risks associated with pregnancy hypertension, as well as awareness-raising and education about birth preparedness and complication readiness (e.g., prior permissions for care-seeking, transport plans, and funding care of obstetric emergencies).

In intervention areas, the LHWs were encouraged to provide domiciliary visits every four-weeks (<28 weeks), fortnightly (28–35 weeks), weekly (≥36 weeks until birth), within 24 h of birth, and on postpartum days 3, 7, and 14. Given the nature of tasks performed by LHWs, these visits were not mandated by LHW supervisors. Tasks were guided by the tablet-based PIERS On the Move (POM) mobile health (mHealth) application (app) [16], that included the miniPIERS (Pre-eclampsia Integrated Estimate of RiSk) time of disease risk stratification [17] with pictograms as visual prompts. Altogether 223 LHWs (and associated 13 Lady Health Supervisors) were trained to provide the CLIP intervention. The three-day didactic and participatory training consisted of BP measurement and use of the CLIP POM tool, with feedback and reinforcement intervention provided at regular monthly meetings.

In brief, the POM directed LHWs to first observe women to rule out emergency conditions (illustrated by pictograms) that would warrant immediate referral to a facility. In the absence of emergency conditions, LHWs were directed to measure BP twice, using standardised methods and a semi-automated digital device validated for use in pregnancy (Microlife BP 3AS1-2) [18]; a third measurement was required if either the systolic or diastolic BP differed by more than 10 mmHg between the first two readings. From January 2015 to December 2016, LHWs also used a pulse oximeter to perform peripheral capillary oxygen saturation (SpO_2_) at the first and any subsequently hypertensive visit [19].

The CLIP POM tool stratified women into one of three care pathways: usual ante-/postnatal care, non-urgent referral to a comprehensive emergency obstetric care (CEmOC) or higher facility within 24 h, or urgent referral to a CEmOC or higher facility within four hours. In addition, women could be recommended to receive oral antihypertensive therapy (for severe hypertension) or intramuscular magnesium sulphate (for evidence of pre-eclampsia) (Fig. S1). In control clusters, women received routine ANC provided at PHCs by nurses and doctors and additional routine LHW domiciliary visits.

During 2015, health care providers from all secondary and tertiary, public and private facilities in the districts received three professional development sessions immediately prior to launching the trial and twice during the first year, focussed on evidence-based detection and management of pregnancy hypertension.

### 2.4 Baseline survey and surveillance

In intervention and control clusters, 20 LHW programme-independent surveillance teams (140 individuals) were trained to undertake quarterly cross-sectional visits. Each team consisted of one male team leader (chaperone) and six female data collectors who visited all households and screened for married, pregnant women aged 15–49 years. Following informed consent, baseline individual- and household-level data were collected (Table 1). At each visit registered women were asked about antenatal care-seeking and birth preparedness, and postpartum, about adverse maternal (<42 days postpartum), fetal, or neonatal events (<28 days). When maternal or perinatal deaths were identified, an independent, experienced team was notified to perform verbal and social autopsies.

**Table 1:** Baseline characteristics.

	Intervention (n = 20,235 pregnancies)	Control (n = 19,182 pregnancies)
**Clusters**	10	10
Population density (n/ha) *	4·1	5·3
Estimated annual birth rate/cluster (n/1000)	22	22
LHWs/cluster	21 [17,30]	21 [13, 21.8]
Neonatal mortality ratio/1000 live births (in previous 12 months at baseline) *	27 [25·1, 36·8]	28·3 [20·7, 34·1]
Households	16,373	15,569
**Enrolled pregnancies**	20,235	19,182
Nature of respondent was woman herself	19,737 (97·5%)	18,612 (97·0%)
Maternal age (year) *	28 [25,30]	28 [25,30]
Married	20,186 (99·8%)	19,109 (99·6%)
Religion		
Muslim	16,806 (83·1%)	15,637 (81·5%)
Hindu	3374 (16·7%)	3472 (18·1%)
Other	18 (0·1%)	5 (0·02%)
Women with ≥ 5 years of schooling *	3971 (19·6%)	3277 (17·1%)
Husbands with ≥ 5 years of schooling *†*	9532 (47·2%)	8247 (43·2%)
**Obstetric history**		
Parous *	15,540 (77·0%)	14,981 (78·4%)
Parity	2 [1,4] (missing 0·3%)	2 [1,4] (missing 0·4%)
Amongst previously pregnant women		
Had previous stillbirth(s) *‡*	2368 (14·6%) (missing 11·4%)	2276 (14·6%) (missing 11·2%)
Had previous neonatal death(s)	2318 (15·2%) (missing 0·03%)	2263 (15·4%) (missing 0·0%)
Delivery location in previous pregnancy *†*		
Home	5051 (31·1%)	5301 (34·1%)
CEmOC (hospitals)	7813 (48·0%)	7522 (48·4%)
Non-CEmOC facility	3342 (20·6%)	2645 (17·0%)
ANC care sought **	13,375 (72.5%)	14,153 (81.6%)
**Current pregnancy**		
Gestational age at enrolment (week)	19·8 [14·1, 26·8]	20·6 [14·9, 27·4]
Multiple pregnancy	171 (0·8%)	182 (0·9%)

Data presented as median [interquartile range] or number (%). * Variables used as adjustment factors in analyses, chosen *a priori*. *†* Variable used as adjustment factor in analyses, chosen following review of baseline data prior to knowledge of outcomes. *‡* Not asked in pilot phase. ** ANC care sought includes only visits with a mandated blood pressure measurement, excluding LHW CLIP contacts and all other LHW contacts.

LHW = Lady Health Worker. CEmOC = Comprehensive emergency obstetric care.

Data collection tools were developed iteratively with local investigators, derived from existing validated questionnaires where possible (e.g., WHO 2012 Verbal and Social Autopsy,[20] and translated to Sindhi (local language). Data were collected initially on paper forms (pilot phase) and then electronically using tablets (definitive phase). Data management protocols ensured data security by encryption, data tracking through user identification numbers and audit trails, and effective data synchronisation between within-cluster devices and the REDCap server (Vanderbilt University, USA) at UBC.

### 2.5 Outcomes (Panel 1)

The primary outcome was a composite of all-cause maternal, fetal, and newborn mortality and major morbidity. Pregnancies with multiple elements of the primary outcome were only counted once and no weighting was used. In addition to stillbirths, maternal and newborn deaths were estimated until 42 and 28 days after birth respectively. Maternal morbidity consisted of serious end-organ complications that included, but were not limited to, those related to pregnancy hypertension. Neonatal morbidity reflected problems related to early delivery or delivery of a baby in poor health. All deaths, as well as survived morbidities, were reviewed on a quarterly basis by an independent panel of physicians, masked to the cluster of origin, and excluding individuals who cared for the woman or baby under review.

The major secondary outcomes were birth preparedness and complication readiness, delivery in a facility able to provide emergency obstetric care, and proportion of facility births. Other outcomes included pre-eclampsia knowledge and awareness before birth, gestational age at birth, and mode of delivery.

### 2.6 Sample size and randomisation

Assuming an annual birth rate of 14/1000/year in each cluster, a baseline incidence of maternal and perinatal mortality and morbidity of 9·6% in control clusters, 10% loss to follow-up, an intra-cluster co-efficient (ICC) of 0·002, and a 20% reduction in all cause maternal, fetal, and newborn morbidity and mortality, an alpha of 0·05, and 80% power, we calculated that we would require 20 clusters over two years. The data upon which the estimates were based on the published or available ranges and were provided by the site investigators based on recent surveillance data. A predetermined review of these sample size assumptions using data from the CLIP feasibility study, pilot and definitive trials was conducted independently by the statistical team (JB, TL, HW, JS) at study midpoint.

The unit of randomisation (cluster) was a Union Council (average population 32,000), and all associated villages and primary health centres (PHCs). The local team chose potential clusters based on comparability of health care infrastructure, surveillance team accessibility, and the absence of conflicting concurrent research activity. Restricted, stratified randomisation was undertaken according to population size. There were 3424 possible allocations of clusters to intervention or control, from which one was randomly selected. Two intervention and control clusters were chosen for the pilot phase based on their implementation readiness.

### 2.7 Statistical analysis (full plan, Appendix 2)

All pregnancies, except those of women who withdrew, were included in our primary, intention-to-treat analyses. The unit of analysis was pregnancy, classified as ‘followed-up’ (complete postpartum trial surveillance), ‘lost-to-follow-up’ (estimated date of delivery [EDD] at ≥ 3 weeks before trial end but without follow-up data), and ‘still-on follow-up’ (EDD < 3 weeks before trial end).

To mitigate potential bias due to differential loss-to and incomplete follow-up, the primary outcome of women who were lost-to, or still-on, follow-up was imputed ten times via multiple imputation by chain equations and Rubin’s rules [21]. Imputation models were based on all primary analysis adjustment factors (see below) and interactions between trial arm and enrolment date (accounting for possible lag in intervention effects). In each imputed data set, the adjusted odds ratio (OR) for the intervention effect was estimated using a multi-level logistic regression including a random intercept term for each cluster. To improve precision, models were adjusted for variables at individual (i.e., age, parity, maternal primary education, previous delivery locations, and husband’s primary education) and cluster-level (i.e., baseline neonatal mortality rate, LHW density, and population density). Sensitivity analysis including only complete cases was conducted with the same adjustment variables.

Further, analogous multi-level logistic regression models were fit to assess the sensitivity of the primary result to various other factors, including adjustment, missing data for a component of the primary outcome (when others were documented), gestational age at birth, and postpartum follow-up to < 42 days, as well as cluster-level aggregate analysis. Where sensitivity analyses included imputation, results were pooled (Rubin’s rules) [21]. In an additional planned secondary analysis, we explored within the intervention arm, whether there was a relationship between our primary outcome and the intensity of CLIP contacts, categorised as 0, 1–3, 4–7, or ≥8, to reflect prior and current WHO recommendations for the frequency of antenatal contacts [22]; to account for factors related to the number of POM-guided visit and confounders, the analysis was adjusted for maternal age, basic education, parity, enrolment timing in the trial, and distance from the household to facility.

All analyses were repeated for each component of the primary outcome, albeit without imputation. Secondary and other outcomes were compared by baseline factor-adjusted multi-level models, as above. Statistical significance (two-sided) was set at p < 0·05 for the primary and p < 0·01 for other analyses, without adjustment for the interim analysis. R statistical software was used throughout.

An interim analysis was undertaken once complete data were received for women making up half of the planned sample size and reviewed by the data safety and monitoring board (DSMB). The stopping rule for both benefit and harm required an observed difference between groups associated with an alpha < 0·001 (power 80%). The DSMB reviewed all reported adverse events for participant safety.

## 3 Results

**Fig. 2:**
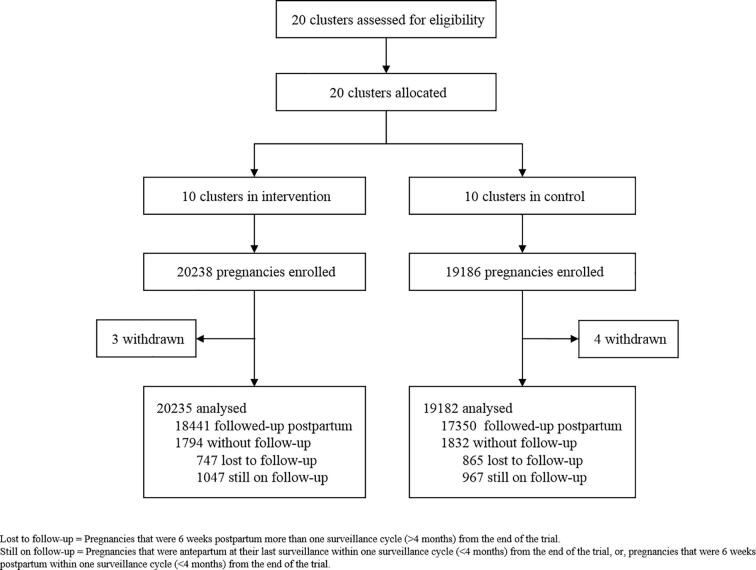
Trial profile – Intervention vs. control allocation clusters.

Between 7 February 2014 and 30 September 2016, 35,974 women (39,417 pregnancies) were recruited in 10 intervention (20,238 pregnancies) and 10 control (19,186 pregnancies) clusters. Of these pregnancies, 4357 (2231 in the intervention vs. 2126 in the control arm) were from the pilot phase. After accounting for 7 withdrawals, 20,235 and 19,182 pregnancies in intervention and control clusters, respectively, were included in the analysis (Fig. 2).

Pregnancies in intervention and control clusters were broadly similar at baseline, with most information coming directly from the participating woman herself (Table 1, Table S2). Women were generally in their late 20s; virtually all were married (some were either widowed or divorced between conception and recruitment) and self-identified as Muslim. Most had no basic education although almost half of their husbands did. Women were generally parous and when so, reported high rates of prior stillbirth (≈15%) and neonatal death (≈15%) (Table S2). Few women (<1%) had multiple pregnancies. Generally, women were enrolled at ≈20 weeks’ gestation (Table 1).

LHW coverage varied widely between clusters (range: 9% (Odero Lal Station) to 98% (Bhanoth)). There were 1368 community engagement sessions/cluster (total of 793 men’s group sessions (7784 participants) and 16,691 LHW-led sessions in pregnant women’s homes).

The visit number compliance target of 12 visits during pregnancy and the post-natal period was met in 5723 (28.3%) pregnancies. Of the total 58,174 visits, 10,529 women (11,399 pregnancies [56·6%]) received ≥ 1 LHW-provided POM visit, for a median of 5 [IQR 3, 7] visits per pregnancy when received. Most visits were antenatal (40,264 [69·2%]). Compliance with BP measurement at all visits, and proteinuria testing during first and all hypertensive visits, was almost uniform (99·9% and 98·4%, respectively).

**Supplementary figure 2:**
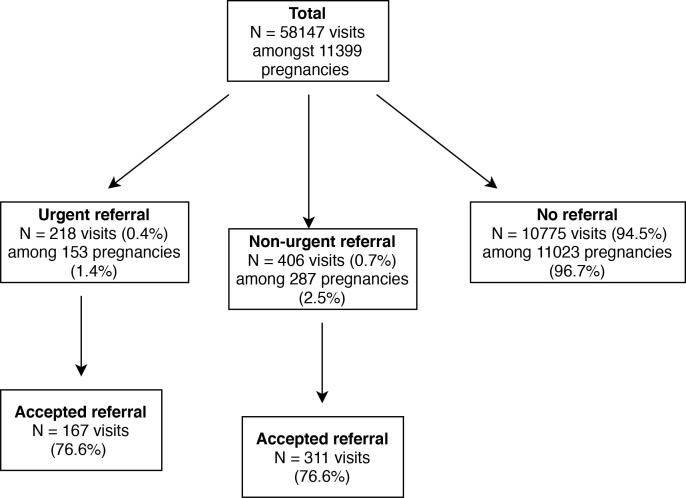
Details of referral type according to CLIP triggers and LHW actions.

POM-guided advice for referrals, whether non-urgent (2·5% of pregnancies) or urgent (1·4% of pregnancies), were accepted 77% of the time. Non-urgent referral (2·5%) was for non-severe hypertension. Urgent referral (1·4%) was most commonly for severe hypertension (153/218, 70·2%) or absent fetal movements in the preceding 24 h (24/218, 11·0%), and accepted 84·9% of the time. Intramuscular magnesium sulphate (for pre-eclampsia) was recommended in 166 (0·3%) of POM visits and accepted in 96 visits (58·3% of the time). Oral methyldopa (for severe hypertension) was recommended in 153 (0·3%) visits and accepted in 148 visits (96·8% of the time). Details of the indications for referral according to the CLIP triggers, and LHW actions, can be found in Table S3 and Figs. S1 and S2.

There was no significant difference in ≥4 ANC visits for women in intervention (44·6%) vs. control (31·7%) clusters (aOR 1·84 95% CI [0·89-3·83]; p = 0·032).

The composite primary outcome (one or more element) occurred in more than 20% of pregnancies, related primarily to major maternal morbidity and fetal/newborn death (≈10% each) (Table 2). The primary outcome did not differ between intervention and control clusters overall (aOR 1·20 [0·84–1·72]; p = 0·31) (Table 2), or by cluster (Table S4). Major maternal morbidity was 37 times more common than was maternal death (2·7/1000 identified pregnancies). Stillbirths (≥20 weeks) and neonatal death each complicated ≈5% of pregnancies. Overall, there were no significant differences between the groups for the most common major maternal morbidities (sepsis (6.8%), followed by antepartum haemorrhage (2·9%) and blood transfusion (1·0%)). The most common major neonatal morbidity was feeding difficulty (3·1%), followed by lethargy (3·0%) and hypothermia (1·8%).

**Table 2:** Primary outcome.

	Intervention (n = 20,235 pregnancies)	Control (n = 19,182 pregnancies)	Adjusted OR*	p-value
Pregnancies with postpartum surveillance	18,419	17,335		
Infants	18,543	17,488		
**Composite primary outcome***†*	5373 (26·6%)	4187 (21·8%)	1·20 [0·84–1·72]	0·31
Maternal mortality	55 (0·3%)	51 (0·3%)	1·08 [0·69–1·71]	0·74
Maternal morbidity	2196 (10·9%)	1707 (8·9%)	1·12 [0·57–2·16]	0·77
(including maternal deaths)				
Obstetric sepsis	1477 (7.3%)	1191 (6·2%)		
Antepartum haemorrhage	643 (3.2%)	487 (2·5%)		
Blood transfusion	240 (1.2%)	168 (0.9%)		
Stroke	58 (0.3%)	79 (0·4%)		
Fistula	56 (0.3%)	39 (0·2%)		
Seizure	32 (0.2%)	18 (0·1%)		
Maternal coma	27 (0.1%)	25 (0·1%)		
Cardiopulmonary resuscitation	11 (0.1%)	17 (0·1%)		
Mechanical ventilation	11 (0·1%)	9 (0·0%)		
Disseminated intravascular coagulation	4 (0·0%)	8 (0·0%)		
Dialysis	2 (0·0%)	3 (0·0%)		
Interventions for major postpartum haemorrhage	2 (0·0%)	5 (0·0%)		
Perinatal mortality and late neonatal death	1942 (9·6%)	1906 (9·9%)	0·95 [0·86–1·03]	0·22
Stillbirth	935 (4·6%)	951 (5·0%)	0·89 [0·81–0·99]	0·03
Early neonatal death	819 (4·0%)	818 (4·3%)	0·95 [0·82–1·09]	0·46
Late neonatal death	197 (1·0%)	150 (0·8%)	1·23 [0·97–1·55]	0·09
Neonatal morbidity	2012 (11%)	1250 (7·2%)	1·22 [0·77–1·96]	0·40
Neonatal morbidity (excluding deaths)				
Feeding difficulty	844 (4·2%)	385 (2.0%)		
Lethargy	585 (2.9%)	609 (3.2%)		
Hypothermia	405 (2.0%)	288 (1.5%)		
Jaundice	385 (1.9%)	389 (2.0%)		
Breathing difficulty	273 (1·3%)	223 (1.1%)		
Seizure	253 (1.3%)	261 (1.4%)		
Umbilical cord infection	284 (1·4%)	241 (1.3%)		
Coma	149 (0.7%)	109 (0.6%)		
Bleeding	82 (0·4%)	44 (0.2%)		
Central nervous system-related morbidity	12 (0·1%)	14 (0.1%)		
Skin infection	2 (0·0%)	1 (0·0%)		

Data presented as number (%) or number only. *Adjusted odds ratio presented as odds ratio [95% confidence interval]; adjusted for individual-level factors (maternal age, parity, maternal education, marital status, husband’s education, delivery location), and cluster-level factors (population density, baseline study neonatal mortality rate, healthcare worker density). † Defined as one/more of maternal morbidity or mortality, stillbirth, neonatal mortality, or neonatal morbidity.

OR = odds ratio.

There was no difference in intervention (vs. control) clusters for birth preparedness (42·4% vs. 29·1%, respectively; aOR 2·41 [0·67-8·67]; p = 0·08) or pre-eclampsia knowledge and awareness (16·8% vs. 14·7%, respectively; aOR 1·11 [0·33-3·69]; p = 0·83).

**Supplementary figure 3:**
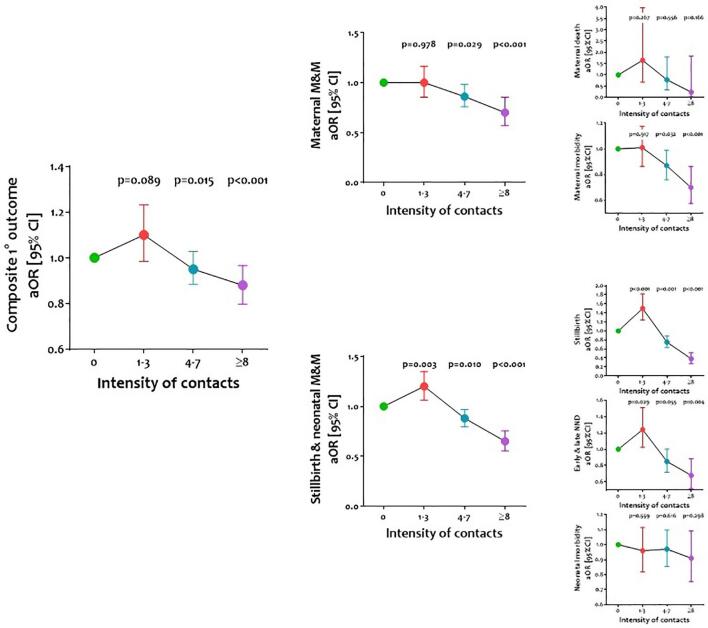
Relationship between the intensity of POM-guided contacts per pregnancy and outcomes in intervention arm women.

Within intervention clusters, women who received both 4–7 and at least eight POM guided contacts experienced fewer adverse outcomes compared with women who received no contacts (28·8% (4–7) and 23·2% (≥8) vs 30·5% (0), respectively (aOR 0·89 [95% CI 0·81-0·98]; p = 0·015 and 0·66 [95% CI 0·58-0·76]; p < 0·001, respectively) (Table 3; Fig. S3). Women with 1–3 POM-guided contacts did not have apparent benefit compared with those without POM contacts (Table 3; Fig. S3). Rates of stillbirth were higher in the 1–3 contact group (7·4% vs 5·7%; aOR 1·50 [95% CI 1·24–1·82]; p < 0·001), with a trend to more neonatal deaths (p = 0·029), compared with those receiving no contacts (Table 3; Fig. S3).

**Table 3:** Relationship between intensity of POM-guided CLIP contacts and the primary outcome.

Outcomes	Number of POM-guided visits										
	0 visits		1–3 visits			4–7 visits			≥8 visits		
	Event rate	Adjusted OR (95% CI) †	Event rate	Adjusted OR (95% CI) †	p	Event rate	Adjusted OR (95% CI)†	p	Event rate	Adjusted OR (95% CI)†	p
Primary outcome‡	2292 (30·5%)	*Reference*	851 (33·4%)	1·10 [0·99–1·23]	0·09	1726 (28·8%)	0·89 [0·81–0·98]	0·015	419 (23·2%)	0·66 [0·58–0·76)	<0·001
Maternal outcome	854 (11·4%)	*Reference*	369 (14·5%)	1.00 [0·85–1·16]	0·98	767 (12·8%)	0·86 [0·76–0·99)	0·029	177 (9·8%)	0·70 ]0·57–0·85]	<0·001
*Maternal mortality*	17 (0·2%)	*Reference*	9 (0·4%)	1.65 [0·68–3·98]	0·27	10 (0·2%)	0·78 [0·33–1·81)	0·56	1 (0·1%)	0·23 [0·03–1·84]	0·17
*Maternal morbidity*	842 (11·2%)	*Reference*	367 (14·4%)	1.01 [0·86–1·18]	0·92	761 (12·7%)	0·87 [0·76–0·99]	0·032	177 (9·8%)	0·70 [0·58–0·87]	0·001
Fetal or neonatal adverse outcome	1801 (24·0%)	*Reference*	651 (25·6%)	1·20 [1·06–1·35]	0·003	1204 (20·1%)	0·88 [0·79–0·97]	0·010	297 (16·4%)	0·65 [0·55–0·75]	<0·001
*Stillbirth*	425 (5·7%)	*Reference*	189 (7·4%)	1·50 [1·24–1·82]	<0·001	271 (4·5%)	0·75 [0·63–0.89)	0·001	50 (2·8%)	0·38 [0·28–0·52]	<0·001
*Neonatal mortality*	448 (6·3%)	*Reference*	178 (7·5%)	1·24 [1·02–1·51]	0·029	308 (5·4%)	0·85 [0·72–1·00]	0·055	77 (4·4%)	0·68 [0·52–0·89]	0·004
*Neonatal morbidity*	1080 (14·4%)	*Reference*	343 (13·5%)	0·96 [0·82–1·11]	0·56	742 (12·4%)	0·97 [0·86–1·10]	0·62	209 (11·6%)	0·91 [0·75–1·09]	0·30

CI = confidence interval. OR = odds ratio.

A single adverse event was reported due to administration of the intervention to a non-pregnant woman who had not been enrolled (Table 4). The event was identified promptly, and trial activities paused during steering committee review of events. The DSMB concluded that no harm resulted from these actions.

**Table 4:** Secondary, safety, and other outcomes.

	Intervention (n = 20,235 pregnancies)	Control (n = 19,182 pregnancies)	Adjusted OR*	p-value
**Secondary outcomes**				
Birth preparedness and complication readiness †	8532 (42·2%)	5580 (29·1%)	2·41 [0·67-8.67]	0·077
Proportion of facility births	13,517 (66·8%)	12,709 (66·3%)	0·96 [0·71-1·29]	0·695
Birth at a CEmOC facility	3949 (65·5%)	3645 (68·3%)	0·9 0·60-1·34]	0·482
**Other outcomes**				
Safety outcomes				
SAEs unrelated to intervention	0 (0·0%)	0 (0·0%)	NA	NA
Adverse events				
Transport-related injury or death	0/305 (0·0%)	NA	NA	NA
Injection site haematoma/infection after community administration of IM MgSO4	0/73 (0·0%)	NA	NA	NA
Injection site complications after any administration of IM MgSO4	NA	NA	NA	NA
Respiratory depression, coma or death during transport following in-community MgSO4	NA	NA	NA	NA
Maternal sBP < 110 mmHg on facility arrival following in-community methyldopa	NA	NA	NA	NA
Deliveries (all birth outcomes)	18,418	17,331	–	–
Miscarriage**	559 (3·0%)	500 (2·9%)	1·10 [0·78–1·57]	0·476
Livebirth	16,943 (83·7%)	15,913 (83·0%)	1·02 [0·87–1·21]	0·708
Deliveries (excluding miscarriage)	17,862	16,835		
Gestational age at delivery(week)	38·7 [36·0–41·0]	38·6 [35·7–41·0]		0·883
Deliveries < 37 weeks	5475 (29·7%)	5543 (32·0%)	0·96 [0·72–1·29]	0·752
Deliveries < 34 weeks	2432 (13·2%)	2466 (14·2%)	0·93 [0·73–1·19]	0·450
Missing	1112 (6·0%)	937 (5·4%)		

Data presented as median [interquartile range] or number (%) or number only. *Odds ratio adjusted for individual-level (i.e., maternal age, parity, maternal primary education, previous delivery locations, and husband’s primary education) and cluster-level (i.e., baseline neonatal mortality rate and population density characteristics). †Birth preparedness and complication readiness was defined as an answer to ALL three of the following: 1) arranged for transport, 2) obtained prior permission to seek emergency care, and 3) saved money for obstetric care.

OR = odds ratio. CEmOC = comprehensive emergency obstetric care. sBP = systolic blood pressure.

## 4 Discussion

In the primary, pre-defined analysis, the CLIP Pakistan Trial intervention did not have a significant impact on either the composite outcome of maternal, fetal, newborn mortality and severe morbidity, or individual components thereof. This was an effectiveness trial with functional public sector LHWs, who despite busy workloads were able to undertake the recommended number of home visits in nearly half the cases. The intervention was reasonably well received by families though the overall reported morbidities detected by POM were low.

The finding of a positive effect of contact intensity of at least four contacts, and more so with at least eight contacts, provides data to support both the previous and current WHO recommendations.[22] While the current WHO recommendations are based on improved perinatal outcomes, our findings (vs. 0 contacts) imply both maternal and perinatal outcomes improved (Table 3 and Fig. S2). Women with 0 contacts were likely to live closer to facilities than those with 1–3 contacts; these latter women had higher rates of fetal and neonatal mortality. Compared with women who received 1–3 contacts, other than maternal death and neonatal morbidity, all components of the primary outcome were significantly reduced with at least four contacts.

Our study design and operational plan had several strengths. We successfully engaged civil society, from women themselves, their families, faith leaders, and government ministries. Complementing the existing health care system by task-sharing, the LHWs used the POM mHealth platform to conduct independent antenatal and postnatal visits and collect their findings. Our community-based data collection procedures resulted in a low (<5%) loss to follow-up despite enrolment of nearly 40,000 pregnancies. However, including only married women with settled addresses excluded single and internally displaced women at higher risk of adverse outcomes; the trial was conducted following the significant internal displacement related to the 2010 Indus River floods.

Notwithstanding the careful design of the intervention, the effect could have been limited by a number of factors. There were insufficient LHWs to deliver the intervention to reach all sections of the population, as demonstrated by <80% of women receiving a POM visit and <80% compliance with the pre-specified schedule of visits when they occurred. The LHWs have significant tasks related to participation in the periodic polio eradication campaigns which overall took almost a third of their time. The annual community engagement may not have been sufficient to induce awareness and behaviour change. We had based our threshold for treatment and referral due to systolic hypertension to increase generalisability where functioning sphygmomanometers and training are lacking; in retrospect, this may have limited the impact of the CLIP intervention as CLIP data have shown that non-severe hypertension was predominately isolated diastolic hypertension [23]; the advent of low-cost, validated (in pregnancy and pre-eclampsia), semi-automated BP devices has mitigated some of the BP measurement concerns [18]. However, despite acceptance and ready application in training programmes, the lack of impact of three technological innovations (BP measurement device, POM and pulse oximetry) also raises the question of the role of technology in increasing community engagement and effectiveness of these frontline workers. The fact that there was no impact on knowledge and awareness of pre-eclampsia and danger signs among participating women suggests that these domiciliary interactions by LHWs may not have been effective. Finally, despite recruiting almost 36,000 women (and almost 40,000 pregnancies), we may have been underpowered to find a difference between trial arms as power is determined by cluster number and the between-cluster outcome rate variability was greater than anticipated (i.e., ICC 0·044).

We undertook community-ascertainment of outcomes. While measures of self-reported maternal, fetal, and neonatal outcomes have been validated in other studies [24], misreporting may have occurred. Also, the process of community engagement may have sensitised intervention cluster women and their families to disproportionately report adverse events which could explain the higher reported event rates in the intervention clusters in the first surveillance quarter. We were unable to use physical examination or chart review to confirm diagnoses, by virtue of the personnel who provided care (i.e., LHWs) and the location of that care (i.e., in the community where there is no health record). However, to mitigate this, a group allocation-masked physician review to adjudicate every reported maternal, fetal or newborn death or major morbidity, was performed. Recall bias risks were minimised by conducting quarterly surveillance cycles.

Our population-based estimates of maternal, fetal, and neonatal mortality tended to be higher than those previously published for Pakistan. Most previous estimates for Pakistan have been facility-based. Our maternal mortality estimate was 323/100,000 livebirths (compared with 259 (AMANHI cohort of 31,114 pregnancies in Matiari) [25] and 299 (secondary analysis of the WHO Multicountry Survey 2011) [26]. For stillbirths, our estimate was 57·5/1000 livebirths (compared with 42·8 and 27·6/1000 livebirths for Matiari and nationally, respectively).[2], [25] Our estimate of neonatal mortality was 60·4/1000 livebirths (compared with 46·9 and 55/1000 livebirths for Matiari and nationally [Pakistan DHS 2011–12], respectively [27]. For stillbirths, these differences may have reflected more comprehensive and accurate ascertainment of outcomes that may have been previously misclassified as miscarriages in the community, and that may not have come to the attention of those providing facility-based care.

The comparability of our community-ascertained rates of morbidity are more difficult to assess, as comparable published data are limited and facility-based. Our incidence of maternal sepsis (7·4%) was much higher than the recorded incidence of sepsis recorded in tertiary facilities in Punjab and Sindh (1·3% in all women) [28]; however, this definition of sepsis (comprising abortion-related infection, puerperal endometritis, pyelonephritis, influenza-like illness, other systemic infections) excluded organ dysfunction whereas our broad definition of sepsis (fever and one of: abdominal/uterine tenderness, foul smelling vaginal discharge/lochia, productive cough and shortness of breath, dysuria or flank pain, headache and neck stiffness) could have captured associated complications related to organ dysfunction. The incidence of antepartum haemorrhage in our trial (3·2%) was within the range reported in facility-based studies in Peshawar (3·0%) [29] or Hyderabad (5·4%). We found no comparable regional data on population level estimates of neonatal morbidities.

A crucial factor limiting the effect on mortality and severe morbidity could also be the design which did not include facility-based interventions and care. Our hypothesis was that implementing community-level evidence-based care would reduce hypertension-related (and potentially, all cause) maternal and perinatal mortality and major morbidity, by addressing the ‘three delays’ in triage, transport, and treatment. While we did undertake staff capacity enhancement throughout the district, we did not specifically put measures in place to improve quality of care. Poor-quality facility care and availability of key commodities could have limited the beneficial effect of community-level intervention on outcomes and negatively influenced community demand; perceived poor quality of care at public hospitals is a significant barrier to seeking care [4], and health care providers in Sindh have gaps in their knowledge about the aetiology, diagnosis, and treatment of pre-eclampsia [6].

Finally, while the individual components of the complex CLIP intervention were evidence-based, how these components interacted with one another and moderated any impact of the intervention remains unclear but will be the subject of pre-determined secondary analyses.

The CLIP trial further confirms the ability of LHWs to task-share important aspects of maternity care, with favourable effects on outcomes, as observed for newborns and children with other community-level interventions in Pakistan [13], [30]. For example, neonatal mortality was reduced by LHW-guided care (basic newborn resuscitation and identification and treatment of suspected neonatal respiratory infections (RR 0·80 [0·68, 0·93]; p = 0·005)) [13] or LHW-led rural group sessions focussed on promotion of antenatal care and maternal health education, use of clean delivery kits, facility births, immediate newborn care, identification of danger signs, and promotion of care seeking (RR 0·85 [0·76, 0·96]; p = 0·02) [30]. Other community-level interventions in Pakistan have noted similar non-specific temporal improvements to those that we observed, and also highlighted the limitation of technology- and home-based care strategies for reducing newborn mortality when separated from facility enhancement [13].

## 5 Conclusions

Pakistan is striving to meet global targets for the sustainable development goals to improve maternal, fetal, and newborn outcomes while tackling the challenge of limited human resources, especially skilled medical professionals in its rural and remote populations. This was the genesis of our study to evaluate what could be done to mitigate the morbidity and mortality associated with pre-eclampsia. The CLIP Pakistan Trial demonstrates that an existing cadre of public sector community health workers can use technology to provide accurate and objective referral advice in pregnancy and engage and mobilize communities. However, as implemented, the intervention did not improve outcomes. To achieve benefits on mortality and severe morbidity, these efforts must be complemented with facility-based strengthening of quality clinical care and facilitation of community-to-facility referral to achieve maximal impact on the unacceptably-high burden of death and morbidity in such settings.

## Contributors

RQ, ZAB, PvD, LAM, BAP, JMA, AS, HN conceptualised the Trial and the components of the intervention. ZAB, RQ, SS, ZH, BAP, MV, SS co-ordinated activities and monitoring of the Trial. IA, JL, TL, DT, DD were responsible for data collection, transfer and data cleaning. JS, HW, TL, JB performed the analyses. PvD, LAM, SS wrote the first draft of the manuscript. All authors provided feedback and review of the manuscript.

## Declaration of Competing Interest

The authors declare that they have no known competing financial interests or personal relationships that could have appeared to influence the work reported in this paper.
