# Supplementary material for: Community-level interventions for pre-eclampsia (CLIP) in Pakistan: A cluster randomised controlled trial
Source: Pregnancy Hypertens. 2020 Oct;22:109–18. doi: 10.1016/j.preghy.2020.07.011 (PMC7694879; doi:10.1016/j.preghy.2020.07.011)
Supplement: Supplementary data 2 [file mmc2.docx]

**Supplemental tables**

- Table S1: CLIP Pakistan Working Group
- Table S2: Baseline characteristics by cluster
- Table S3: CLIP triggers and treatment
- Table S4: Outcome rates for each cluster
- Table S5: CLIP Intervention
- Table S6: Sensitivity analyses for primary outcome

***Table S1:* CLIP Pakistan Working Group for appearance on PubMed**

| **First and middle names** | **Last names** |
| --- | --- |
| Amjad | Hussain |
| Javed | Memon |
| Farrukh | Raza |
| Sharla K | Drebit |
| Chirag | Kariya |
| Mansun | Lui |
| Diane | Sawchuck |
| Ugochi V | Ukah |
| Mai-Lei | Woo Kinshella |
| Shafik | Dharamsi |
| Guy A | Dumont |
| Tabassum | Firoz |
| Ana Pilar | Betrán |
| Susheela M | Engelbrecht |
| Veronique | Filippi |
| William A | Grobman |
| Marian | Knight |
| Ana | Langer |
| Simon A | Lewin |
| Gwyneth | Lewis |
| Craig | Mitton |
| Nadine | Schuurman |
| James G | Thornton |
| France | Donnay |
| Kelly | Pickerill |

***Table S2:* Baseline characteristics by cluster**

|  | Intervention | | | | | | | | | | Control | | | | | | | | | |
| --- | --- | --- | --- | --- | --- | --- | --- | --- | --- | --- | --- | --- | --- | --- | --- | --- | --- | --- | --- | --- |
| Cluster | Hatri | Moolan | Bhanoth | Shah Alam Shah Ji Wasi | Tando Qaiser | Karam Khan Nizamani | Masu Bhurgari | Odero Lal Station | Odero Lal Village | Haji Sawan Khan Gopang | Muso Khatian | Tando Haider | Tando Fazal | Hossri | Seri | Tajpur | Bau Khan Pathan | Bhit Shah | Faqeer Noohthiani | Sekhat/Khyber |
| Population | 36571 | 27856 | 38465 | 34718 | 35397 | 34346 | 29741 | 29425 | 33466 | 36073 | 33654 | 29242 | 34757 | 33549 | 29599 | 34099 | 30803 | 41898 | 35018 | 34948 |
| Lady health workers | 57 | 35 | 98 | 28 | 97 | 56 | 48 | 9 | 24 | 67 | 80 | 100 | 65 | 69 | 61 | 43 | 53 | 55 | 51 | 60 |
| Neonatal mortality ratio/1000 live births (in previous 12 months at baseline) | 35.64 | 24.69 | 26.43 | 23.15 | 26.2 | 37.17 | 39.15 | 27.63 | 39.91 | 17.41 | 11.54 | 24.05 | 13.09 | 19.64 | 31.19 | 47.83 | 30.41 | 26.25 | 35.07 | 40.49 |
| Households | 1860 | 1051 | 1756 | 1839 | 1948 | 2174 | 1134 | 1586 | 1733 | 1292 | 977 | 1095 | 1277 | 989 | 1490 | 1708 | 1745 | 1768 | 1874 | 2646 |
| Enrolled pregnancies | 2153 | 1240 | 2084 | 2274 | 2564 | 2954 | 1338 | 2030 | 2039 | 1559 | 1149 | 1294 | 1505 | 1174 | 1955 | 2006 | 2092 | 2067 | 2260 | 3680 |
| Woman herself as respondent | 2104/2153 (97.72%) | 1219/1240 (98.31%) | 2082/2084 (99.90%) | 2241/2274 (98.55%) | 2425/2564 (94.58%) | 2859/2954 (96.78%) | 1283/1338 (95.89%) | 2027/2030 (99.85%) | 2020/2039 (99.07%) | 1477/1559 (94.74%) | 1048/1149 (91.21%) | 1118/1294 (86.40%) | 1489/1505 (98.94%) | 1159/1174 (98.72%) | 1909/1955 (97.65%) | 1971/2006 (98.26%) | 1953/2092 (93.36%) | 2060/2067 (99.66%) | 2248/2260 (99.47%) | 3657/3680 (99.38%) |
| Maternal age (median) | 28 (25; 30) | 28 (25; 30) | 28 (25; 31) | 28 (25; 30) | 28 (25; 30) | 27 (25; 30) | 27 (25; 30) | 28 (25; 30) | 29 (26; 30) | 30 (25; 35) | 27 (25; 30) | 28 (24; 30) | 28 (25; 30) | 27 (25; 30) | 28 (25; 30) | 30 (25; 33) | 29 (25; 32) | 28 (25; 30) | 27 (24; 30) | 27 (25; 30) |
| Husband with basic education (>=5 years) | 808/2148 (37.62%) | 595/1231 (48.33%) | 1085/2081 (52.14%) | 992/2270 (43.70%) | 1041/2561 (40.65%) | 1560/2945 (52.97%) | 687/1337 (51.38%) | 1079/2027 (53.23%) | 965/2029 (47.56%) | 720/1557 (46.24%) | 592/1144 (51.75%) | 679/1291 (52.59%) | 481/1498 (32.11%) | 494/1167 (42.33%) | 611/1951 (31.32%) | 753/1988 (37.88%) | 756/2076 (36.42%) | 1235/2063 (59.86%) | 1027/2256 (45.52%) | 1619/3675 (44.05%) |
| Woman with basic education (>=5 years) | 431/2153 (20.02%) | 310/1240 (25.00%) | 424/2084 (20.35%) | 340/2274 (14.95%) | 524/2564 (20.44%) | 575/2954 (19.47%) | 282/1338 (21.08%) | 407/2030 (20.05%) | 401/2039 (19.67%) | 277/1559 (17.77%) | 259/1149 (22.54%) | 402/1294 (31.07%) | 184/1505 (12.23%) | 285/1174 (24.28%) | 221/1955 (11.30%) | 329/2006 (16.40%) | 237/2092 (11.33%) | 503/2067 (24.33%) | 306/2260 (13.54%) | 551/3680 (14.97%) |
| Religion |  |  |  |  |  |  |  |  |  |  |  |  |  |  |  |  |  |  |  |  |
| Muslim | 1822/2153 (84.63%) | 1039/1240 (83.79%) | 1818/2084 (87.24%) | 1865/2274 (82.01%) | 2131/2564 (83.11%) | 2235/2954 (75.66%) | 1275/1338 (95.29%) | 1648/2030 (81.18%) | 1689/2039 (82.83%) | 1284/1559 (82.36%) | 1015/1149 (88.34%) | 1100/1294 (85.01%) | 1239/1505 (82.33%) | 987/1174 (84.07%) | 1783/1955 (91.20%) | 1332/2006 (66.40%) | 1546/2092 (73.90%) | 1749/2067 (84.62%) | 1763/2260 (78.01%) | 3123/3680 (84.86%) |
| Hindu | 317/2153 (14.72%) | 195/1240 (15.73%) | 266/2084 (12.76%) | 403/2274 (17.72%) | 431/2564 (16.81%) | 709/2954 (24.00%) | 62/1338 (4.63%) | 379/2030 (18.67%) | 339/2039 (16.63%) | 273/1559 (17.51%) | 129/1149 (11.23%) | 190/1294 (14.68%) | 261/1505 (17.34%) | 179/1174 (15.25%) | 168/1955 (8.59%) | 655/2006 (32.65%) | 533/2092 (25.48%) | 312/2067 (15.09%) | 493/2260 (21.81%) | 552/3680 (15.00%) |
| Christian | 3/2153 (0.14%) | 0/1240 (0.00%) | 0/2084 (0.00%) | 0/2274 (0.00%) | 0/2564 (0.00%) | 3/2954 (0.10%) | 0/1338 (0.00%) | 0/2030 (0.00%) | 2/2039 (0.10%) | 0/1559 (0.00%) | 1/1149 (0.09%) | 1/1294 (0.08%) | 0/1505 (0.00%) | 1/1174 (0.09%) | 0/1955 (0.00%) | 0/2006 (0.00%) | 0/2092 (0.00%) | 0/2067 (0.00%) | 0/2260 (0.00%) | 0/3680 (0.00%) |
| Other | 6/2153 (0.28%) | 1/1240 (0.08%) | 0/2084 (0.00%) | 2/2274 (0.09%) | 0/2564 (0.00%) | 0/2954 (0.00%) | 0/1338 (0.00%) | 1/2030 (0.05%) | 0/2039 (0.00%) | 0/1559 (0.00%) | 0/1149 (0.00%) | 0/1294 (0.00%) | 0/1505 (0.00%) | 0/1174 (0.00%) | 0/1955 (0.00%) | 1/2006 (0.05%) | 0/2092 (0.00%) | 0/2067 (0.00%) | 1/2260 (0.04%) | 0/3680 (0.00%) |
| Parous | 1632 (75.8%) | 957 (77.18%) | 1614 (77.45%) | 1753 (77.09%) | 1964 (76.6%) | 2258 (76.44%) | 1014 (75.78%) | 1553 (76.5%) | 1566 (76.8%) | 1229 (78.83%) | 907 (78.94%) | 950 (73.42%) | 1158 (76.94%) | 897 (76.41%) | 1521 (77.8%) | 1566 (78.07%) | 1658 (79.25%) | 1659 (80.26%) | 1798 (79.56%) | 2867 (77.91%) |
| Parity (median) | 2.0 [1.0; 3.0] | 2.0 [1.0; 4.0] | 2.0 [1.0; 4.0] | 2.0 [1.0; 4.0] | 2.0 [1.0; 4.0] | 2.0 [1.0; 4.0] | 2.0 [1.0; 4.0] | 2.0 [1.0; 4.0] | 2.0 [1.0; 4.0] | 2.0 [1.0; 4.0] | 2.0 [1.0; 4.0] | 2.0 [0.0; 4.0] | 2.0 [1.0; 4.0] | 2.0 [1.0; 4.0] | 2.0 [1.0; 4.0] | 2.0 [1.0; 4.0] | 2.0 [1.0; 4.0] | 2.0 [1.0; 4.0] | 2.0 [1.0; 4.0] | 2.0 [1.0; 4.0] |
| N women had a previous pregnancy resulted in stillbirth | 284/1695 (16.76%) | 203/996 (20.38%) | 278/1687 (16.48%) | 323/1830 (17.65%) | 173/2072 (8.35%) | 230/2394 (9.61%) | 144/1061 (13.57%) | 314/1625 (19.32%) | 222/1629 (13.63%) | 197/1273 (15.48%) | 179/950 (18.84%) | 153/987 (15.50%) | 140/1204 (11.63%) | 186/935 (19.89%) | 152/1585 (9.59%) | 181/1613 (11.22%) | 314/1700 (18.47%) | 294/1704 (17.25%) | 312/1861 (16.77%) | 365/3005 (12.15%) |
| N women who had a previous neonatal death | 238/1585 (15.02%) | 139/936 (14.85%) | 254/1584 (16.04%) | 201/1716 (11.71%) | 331/1947 (17.00%) | 312/2232 (13.98%) | 133/995 (13.37%) | 250/1520 (16.45%) | 253/1536 (16.47%) | 207/1212 (17.08%) | 99/890 (11.12%) | 152/928 (16.38%) | 97/1138 (8.52%) | 94/873 (10.77%) | 239/1505 (15.88%) | 243/1541 (15.77%) | 289/1621 (17.83%) | 298/1633 (18.25%) | 300/1751 (17.13%) | 452/2830 (15.97%) |
| Delivery location in last pregnancy |  |  |  |  |  |  |  |  |  |  |  |  |  |  |  |  |  |  |  |  |
| Home | 404/1695 (23.83%) | 379/996 (38.05%) | 631/1687 (37.40%) | 425/1830 (23.22%) | 853/2072 (41.17%) | 942/2394 (39.35%) | 229/1061 (21.58%) | 353/1625 (21.72%) | 471/1629 (28.91%) | 364/1273 (28.59%) | 356/950 (37.47%) | 324/987 (32.83%) | 377/1204 (31.31%) | 264/935 (28.24%) | 479/1585 (30.22%) | 493/1613 (30.56%) | 665/1700 (39.12%) | 582/1704 (34.15%) | 708/1861 (38.04%) | 1053/3005 (35.04%) |
| CEmOC (hospitals) | 847/1695 (49.97%) | 430/996 (43.17%) | 897/1687 (53.17%) | 1269/1830 (69.34%) | 920/2072 (44.40%) | 1115/2394 (46.57%) | 600/1061 (56.55%) | 247/1625 (15.20%) | 1065/1629 (65.38%) | 423/1273 (33.23%) | 491/950 (51.68%) | 528/987 (53.50%) | 715/1204 (59.39%) | 458/935 (48.98%) | 705/1585 (44.48%) | 941/1613 (58.34%) | 503/1700 (29.59%) | 542/1704 (31.81%) | 832/1861 (44.71%) | 1807/3005 (60.13%) |
| Non CEmOC | 441/1695 (26.02%) | 183/996 (18.37%) | 153/1687 (9.07%) | 135/1830 (7.38%) | 295/2072 (14.24%) | 331/2394 (13.83%) | 228/1061 (21.49%) | 1024/1625 (63.02%) | 90/1629 (5.52%) | 485/1273 (38.10%) | 102/950 (10.74%) | 132/987 (13.37%) | 93/1204 (7.72%) | 213/935 (22.78%) | 393/1585 (24.79%) | 174/1613 (10.79%) | 520/1700 (30.59%) | 577/1704 (33.86%) | 320/1861 (17.20%) | 140/3005 (4.66%) |

CEmOC=comprehensive emergency obstetric care. CLIP=Community-Level Interventions for Pre-eclampsia. IM=intramuscular. IQR=interquartile range. LHW=Lady Health Worker. POM=PIERS On the Move.

***Table S3*: CLIP triggers and treatment**

|  | **Urgent transport** | **MgSO_4_†**  5g IM in each buttock | **Methyldopa‡** 750mg po | **Non-urgent transport** |
| --- | --- | --- | --- | --- |
| **Maternal risk triggers** |  |  |  |  |
| **sBP ≥160mmHg** (consistent with severe pre-eclampsia) | ● | ● | ● |  |
| **Unconsciousness*** | ● |  |  |  |
| With sBP ≥160mmHg | ● | ● | ● |  |
| **Recent stroke or seizure** | ● | ● |  |  |
| With sBP ≥160mmHg | ● | ● | ● |  |
| **Significant vaginal bleeding** | ● |  |  |  |
| With sBP ≥140mmHg (presumed abruption and severe pre-eclampsia) | ● | ● |  |  |
| **miniPIERS predicted probability ≥25%** | ● | ● |  |  |
| **SpO_2_ <93%** | ● |  |  |  |
| **Fetal risk triggers** |  |  |  |  |
| **No fetal movements in last 12hr** | ● |  |  |  |
| **Heavy proteinuria ≥4+** | ● |  |  |  |
| **None of the 7 triggers and non-severe hypertension** (sBP 140-159mmHg) |  |  |  | ● |

** Unconscious women had to be severely hypertensive before receiving MgSO_4_; in the event that their unconsciousness was due to another cause unrelated to hypertension, such as obstetric sepsis or hypotension due to bleeding.*

*† MgSO_4_ was administered for eclampsia or severe pre-eclampsia, defined as hypertension that was either severe, or associated with stroke, abruption, or a high probability (≥25%) of an adverse maternal outcome within the next 48hr.*

*‡ Methyldopa was adminsitered only for severe hypertension.*

sBP=systolic blood pressure. SpO_2_=oxygen saturation by pulse oximetry.

***Table S4:* Outcome rates for each cluster**

| **Cluster** | Combined primary outcome | Combined maternal outcome | Maternal death | Maternal morbidity | Combined fetal and neonatal outcome | Stillbirth | Early neonatal death | Late neonatal death | Neonatal morbidity |
| --- | --- | --- | --- | --- | --- | --- | --- | --- | --- |
| **Intervention** |  |  |  |  |  |  |  |  |  |
| Bau Khan Pathan | 18.78% | 6.86% | 0.32% | 6.70% | 14.10% | 5.69% | 4.79% | 0.80% | 5.85% |
| Bhit Shah | 18.55% | 6.69% | 0.37% | 6.37% | 14.47% | 5.22% | 3.61% | 0.78% | 6.84% |
| Faqeer Noohthiani | 20.39% | 7.55% | 0.34% | 7.36% | 15.58% | 5.29% | 4.71% | 0.91% | 7.79% |
| Hossri | 12.68% | 2.19% | 0.09% | 2.10% | 11.41% | 4.38% | 3.83% | 0.64% | 3.47% |
| Muso Khatian | 32.18% | 19.61% | 0.09% | 19.51% | 18.11% | 5.07% | 4.88% | 0.94% | 9.01% |
| Sekhat/Khyber | 32.74% | 17.36% | 0.35% | 17.18% | 22.96% | 6.54% | 5.42% | 0.71% | 13.97% |
| Seri | 33.96% | 13.47% | 0.25% | 13.28% | 27.57% | 5.70% | 4.95% | 0.81% | 18.30% |
| Tajpur | 18.89% | 7.72% | 0.12% | 7.60% | 14.75% | 5.47% | 3.92% | 1.09% | 6.22% |
| Tando Fazal | 22.11% | 8.28% | 0.42% | 8.14% | 17.19% | 4.63% | 5.96% | 1.26% | 7.51% |
| Tando Haider | 23.78% | 4.72% | 0.43% | 4.55% | 21.63% | 4.98% | 4.21% | 0.86% | 14.25% |
| **Control** |  |  |  |  |  |  |  |  |  |
| Bhanoth | 41.96% | 20.35% | 0.31% | 20.25% | 28.91% | 4.75% | 4.28% | 0.94% | 21.71% |
| Haji Sawan Khan Gopang | 27.22% | 17.32% | 0.14% | 17.25% | 13.54% | 4.19% | 5.70% | 0.89% | 4.26% |
| Hatri | 24.17% | 9.95% | 0.66% | 9.40% | 17.78% | 4.52% | 4.67% | 1.12% | 8.84% |
| Karam Khan Nizamani | 35.03% | 21.66% | 0.15% | 21.62% | 22.49% | 4.61% | 3.48% | 1.13% | 15.53% |
| Masu Bhurgari | 21.12% | 4.69% | 0.32% | 4.53% | 18.85% | 5.66% | 3.72% | 0.97% | 9.87% |
| Moolan | 30.39% | 9.35% | 0.35% | 9% | 25.63% | 5.89% | 5.37% | 1.30% | 13.68% |
| Odero Lal Station | 34.66% | 7.29% | 0.33% | 7.02% | 30.90% | 5.28% | 5.82% | 0.82% | 20.62% |
| Odero Lal Village | 25.49% | 5.65% | 0.34% | 5.43% | 22.16% | 6.22% | 4.01% | 1.13% | 12.72% |
| Shah Alam Shah Ji Wasi | 16.98% | 3.19% | 0.14% | 3.14% | 15.17% | 5.14% | 4.04% | 0.95% | 7.85% |
| Tando Qaiser | 30.29% | 15.85% | 0.30% | 15.55% | 19.12% | 5.06% | 4.21% | 1.36% | 11.17% |

*Data presented as number (%) only·*

***Table S5*: CLIP intervention**

|  | **Intervention**  **(n=20,235 pregnancies)** |
| --- | --- |
| Community engagement sessions | 17,484 |
| Median community engagement sessions/cluster | 78 [61.25 – 96.75] |
| LHWs trained | 224 |
| POM-guided CLIP contacts | 58174 |
| Median POM-guided contacts/pregnancy | 5.00 [3.00 - 7.00] |
| Antenatal | 3.00 [2.00 - 5.00] |
| Postnatal | 2.00 [1.00 - 3.00] |
| Emergency condition identified (and BP not taken) | 7/15 (46·7%) |
| BP measurement (all contact) | 58111 (99·89%) |
| Proteinuria measurement (of first and any hypertensive contact) | 11719/11905 (98·44%) |
| Complete contacts that resulted in a recommendation | 57925/58174 (99·57%) |
| Pregnancies with ≥1 POM-guided CLIP contact | 11399/20235 (56·3%) |
| Complete contacts | 58156/58174 (99·97%) |
| Pregnancies compliant with POM-guided CLIP contact frequency | 5871 (53·84%) |
| Pregnancies given methyldopa | 95 (0·8%) |
| Accepted | 94 (99·0%) |
| Pregnancies given IM magnesium sulphate | 105 (0·9%) |
| Accepted | 74 (70·5%) |
| Pregnancies referred to facility | 378 (3·3%) |
| Accepted | 315 (83·3%) |

CEmOC=comprehensive emergency obstetric care. CLIP=Community-Level Interventions for Pre-eclampsia. IM=intramuscular. IQR=interquartile range. LHW=Lady Health Worker. POM=PIERS On the Move.

***Table S6*: Sensitivity analyses for primary outcome**

|  | Odds ratio [95% CI] | p-value | Adjusted | Imputed |
| --- | --- | --- | --- | --- |
| Composite primary outcome* | 1·20 [0·84-1·72] | 0·32 | ✓ | ✓ |
| Unadjusted OR | 1·32 [0·95-1·83] | 0·09 | ✗ | ✓ |
| Complete postpartum follow up | 1·23 [0·86-1·75] | 0·26 | ✓ | ✗ |
| Complete responses to all components of primary outcome | 1·23 [0·86-1·74] | 0·26 | ✓ | ✗ |
| EDD + 3 weeks falls within trial timeline | 1·21 [0·85-1·73] | 0·30 | ✓ | ✓ |
| EDD + 3 weeks + 42 days falls within trial timeline | 1·22 [0·85-1·75] | 0·29 | ✓ | ✓ |
| Including only pregnancies with POM visits | 1·07 [0·75-1·55] | 0·70 | ✓ | ✓ |
| Cluster-level aggregate model | 1·03 [0·96-1·02] | 0·16 | ✗ | ✗ |

OR=odds ratio. POM=PIERS On the Move. EDD=estimated date of delivery.

** Defined as one/more of maternal morbidity or mortality, stillbirth, neonatal mortality, or neonatal morbidity*
